# Impact of the eHealth literacy, knowledge and attitudes on COVID-19 prevention behavior among residents in the second year of the COVID-19 pandemic: A cross-sectional study in Anhui Province, China

**DOI:** 10.3389/fpubh.2022.1015803

**Published:** 2022-11-02

**Authors:** Ting Yuan, Xiang Dong Li, Ming Zhang, Xiu Bin Tao, Shu Juan Xu, Huan Liu

**Affiliations:** ^1^Department of Gynecology and Obstetrics Nursing, School of Nursing, Wannan Medical College, Wuhu, China; ^2^Department of Gerontology, Yijishan Hospital, The First Affiliated Hospital of Wannan Medical College, Wuhu, China; ^3^School of Innovation and Entrepreneurship, Wannan Medical College, Wuhu, China; ^4^Department of Nursing, Yijishan Hospital, The First Affiliated Hospital of Wannan Medical College, Wuhu, China; ^5^Department of Blood Purification Centre, Yijishan Hospital, The First Affiliated Hospital of Wannan Medical College, Wuhu, China

**Keywords:** knowledge, attitude, preventive behavior, COVID-19, eHealth literacy, residents, China

## Abstract

**Objective:**

The pandemic of COVID-19 continues to challenge people's health worldwide. In the second year of the pandemic, adherence to COVID-19 preventive behavior is key to continuing efforts to overcome the epidemic. This study aims to assess the COVID-19-related knowledge, attitude, and prevention behavior (KAP) and electronic health literacy (eHealth literacy) among Anhui residents in China.

**Methods:**

From January 30 to March 27, 2021, the cross-sectional study was performed among Anhui residents in China, including 16 cities. An online survey was adopted to assess KAP regarding COVID-19, and eHealth, involving a total of 2,122 citizens. Following informed consent, residents were recruited by convenience sampling. Frequencies and proportions were calculated. Additionally, Mann–Whitney *U* tests were used to analyze the variables. Independent predictors of preventive behavior of COVID-19 were ascertained using a multivariable logistic regression model.

**Result:**

Residents demonstrated good knowledge, positive attitudes, acceptable practices, and good eHealth literacy. Online news and WeChat are the main health information resources. Citizens who had good knowledge, a positive attitude, good eHealth, and did not participate in the online lectures or training COVID-19 were more likely to take preventive measures. Those with poor health, who were male, did not have family members working in health care facilities, and did not work in a face-to-face environment were less likely to take precautions. Compared with a master's degree and above, participants with middle school education level and below took preventive behavior sometimes. Residents who browse the COVID-19 webpage <15 min weekly seldom took preventive actions.

**Conclusion:**

The study showed that in the second year of the COVID-19 pandemic, Chinese residents had adequate knowledge of COVID-19, positive attitudes, appropriate preventive practices, and basic eHealth literacy. To prevent the rebound of the COVID-19 epidemic, the government and health agencies should inform citizens concerning which information channels or websites to use and assist the underprivileged population who lacks basic infrastructure. In addition, increasing the level of knowledge and attitude, enhancing eHealth literacy and the Health Belief Model (HBM), and implementing the Health Code were seen as ways to reinforce adherence to preventive behavior. Targeting men, implementing public awareness campaigns, community engagement strategies, and health education programs are recommended.

## Introduction

The coronavirus disease 2019 (COVID-19) was first reported by officials in Wuhan, Hubei Province, China, in December 2019 (1). Deeply concerned by both the speed and severity of transmission, on March 11, 2020, the World Health Organization (WHO) declared COVID-19 as a pandemic (2). As of February 2, 2021, there were more than 102 million confirmed cases and 2.2 million deaths worldwide (3). The COVID-19 pandemic remains out of control globally (4).

The COVID-19 pandemic has led to the most serious health crisis of the 21st century (5).

People around the world experience higher rates of depression, anxiety, stress, and trauma due to the pandemic (6–8). In addition, public concern and fear may be raised after the loss of more than 2 million lives (9, 10).

To control COVID-19 transmissions, high-quality preventive measures and aggressive actions have been implemented by the government, including lockdowns, mask-wearing, handwashing, infected and contact case tracing, detection, and isolation. As a result of these, the global economy has deeply plunged into recession (11). Countless livelihoods were destroyed, millions of people forcibly displaced, the health system disrupted, and people pushed into poverty (12). Also, the lack of social contact and changes in lifestyle have led to an increase in smartphone addiction, internet addiction, alcohol and cannabis use (6, 13–15). Thus, the plight of vulnerable people has been exacerbated (16).

With the vaccine roll-out, public health advocated vaccination as a preferred method of protection. However, SARS-CoV-2 antibodies haven't been shown to confer durable immunity against reinfections up to now (17, 18). SARS-CoV-2 is an RNA virus. SARS-CoV-2 mutations occurred when RNA viruses encoded genes for surface glycoproteins, resulting in lessening the efficacy of vaccines (19). While a number of previous studies have found that vaccinations reduce participants' compliance with public health measures (20–22). Additionally, Italian studies showed that the behavior or attitudes toward the adoption of most protective behaviors on COVID-19 decreased over time (23, 24). Hence, maintaining compliance with preventive measures remains essential for pandemic control.

People infected with COVID-19 can present as either symptomatic or asymptomatic. Growing evidence suggests that asymptomatic carriers of the SARS-CoV-2 can also transmit the virus (25, 26). It is a challenge to control the disease's spread as asymptomatic individuals are more likely to be out rather than be isolated in their homes, which can pose a significant public health risk (27). Therefore, continual precautions should also be taken to prevent viral transmission.

During the global pandemic, it is reported that many individuals rely on the internet as their major source of health-related information (28). On the other hand, misinformation or conspiracy theories on the internet may interfere with or undermine adherence to prevention guidelines, potentially reducing public protective behaviors against the pandemic (29–32).

In response to the global COVID-19 pandemic, eHealth literacy is critical in disease control strategies, which help the public access health information quickly and accurately and avoid the spread of misinformation and conspiracy theories (33). Prior surveys found that higher scores of eHealth literacy were positively associated with physical exercise, a healthy diet, adherence to infection prevention and control measures, and protective behavioral practices (33–37).

Before herd immunity has been built up by vaccination, we will still coexist with SARS-CoV-2. Public health measures should also be carried out. However, the public precautions against COVID-19 in Nigeria, Ethiopia, Egypt, and China were unsatisfactory based on previous studies (4, 38–40). Understanding the predictors of practice against COVID-19 may aid in the resolution of COVID-19 future pandemics. Therefore, to address existing knowledge gaps, further analysis and research on the correlation between public adherence to preventive measures and knowledge, attitudes, and eHealth literacy during pandemic control is needed. Thus, this study aimed to: (1) evaluate the knowledge, attitude, and preventive practices toward COVID-19 of the Chinese population to date; (2) assess the eHealth literacy and their associations with preventive behaviors, (3) and identify the associated factors of individuals' prevention behaviors.

## Methods and materials

A descriptive cross-sectional survey was performed among the general population of Anhui, China, from January 30 to March 27, 2021. The online self-report questionnaire was used to collect data to investigate knowledge, prevention behaviors, attitudes, and electronic health literacy (eHealth literacy) 1 year after the 2019 novel coronavirus outbreak. The access link was shared between acquaintances, friends, family members, and colleagues *via* WeChat. Participants were chosen through convenience sampling.

### Ethical consideration

This research was approved by the Ethics Committee of the School of Nursing of Wannan Medical College (no. 20200012.10).

### Participants

The study recruited 2,946 residents of Anhui, China. After eliminating the participants with missing or incomplete data, and those who met the exclusion criteria, a total of 2,122 samples were used in the surveys. The subjects consented to participate in this survey by volunteering to complete and submit the questionnaire. The participants could withdraw from the survey at any time.

The inclusion criteria for the participants were: (1) ≥18 years old; (2) community-dwelling Chinese residents; (3) willing to participate in the study; and (4) all the questionnaire response time≥180s. The exclusion criteria for the participants were: (1) diagnosed with a mental disorder; and (2) unable to understand the questions completely. The demographics of the study participants are presented in Table 1.

**Table 1:** Sample characteristics (*N* = 2,122).

**Variables**	**Characteristics**	***N***	**%**
Gender	Male	332	15.65
	Female	1,790	84.35
Living areas	City	1,384	65.22
	Countryside	738	34.78
Education level	Middle school and below	74	3.49
	Senior high school	100	4.71
	Junior college	563	26.53
	Bachelor	1,330	62.68
	Master and above	55	2.59
Marital status	Single	1,016	47.88
	Married	1,013	47.74
	Divorced /widowed	93	4.38
Region	Northern Anhui	155	7.30
	Central Anhui	306	14.42
	Southern Anhui	1,661	78.28
Health status	Bad	429	20.22
	Good	1,693	79.78
Age	18~20	556	26.20
	21~30	712	33.55
	31~40	562	26.48
	41~50	217	10.23
	>=50	75	3.53
Face-to-face working environment	No	748	35.25
	Yes	1,374	64.75
Fear of infection with COVID-19	No	882	41.56
	Yes	1,240	58.44
Have a family member working in a medical institution	No	775	36.52
	Yes	1,347	63.48
Browse the COVID-19 webpage time weekly	<15 min	706	33.27
	15–30 min	726	34.21
	30–60 min	271	12.77
	>60 min	419	19.75
Attend online COVID-19 lectures or training	No	589	27.76
	Yes	1,533	72.24
Vaccination with COVID-19	No	1,719	81.01
	Yes	403	18.99
eHealth literacy	Low	520	24.51
	High	1,602	75.49
Exposure to suspected and confirmed COVID-19 patients	No	2,010	94.72
	Yes	112	5.28

Anhui Province is situated in the eastern part of China. The total area of the province is over 139,000 square kilometers, with a population of about 60 million. It is bounded by the provinces of Hubei, Jiangsu, Zhejiang, Jiangxi, Henan and Shandong. Sixteen cities in Anhui Province are defined by administrative divisions. Northern Anhui refers to the area north of the Huai River in Anhui, including the six cities of Suizhou, Huabei, Bengbu, Fuyang, Huainan, and Bozhou. Anhui central refers to the area north of the Yangtze River in Anhui Province, south of the Huai River, including the four cities of Hefei, Lu'an, Chuzhou, and Anqing. Southern Anhui refers to the area south of the Yangtze River in Anhui Province, including the six cities of Huangshan, Wuhu, Maanshan, Tongling, Xuancheng, and Chizhou.

### Questionnaire development

The online survey is based on the eighth edition of the guidelines from the National Health Commission of China and some previous surveys (41–44).

Two public health researchers formed a panel to develop the questionnaire. The questionnaire was tested among 30 Chinese residents to assess clarity, readability, and length. In the pilot study, all the residents shown that the questions were easy to understand, and the length was appropriate and acceptable. These 30 Chinese residents did not participate in the actual survey.

### Questionnaire

The present design of the self-administered structured questionnaire contains 48 questions and consists of five sections.

Section A recorded the participant's demographic data, including sex, living areas, education level, marital status, region, current health status, age, working directly with people face-to-face, fear of infection with COVID-19, family members who work in health care facilities, browsing the COVID-19 webpage time weekly, online COVID-19 lectures or training, vaccination with COVID-19, eHealth literacy, and exposure to suspected and confirmed COVID-19 patients.

Section B gathered information on participants' knowledge of COVID-19 by using 14 items. This section includes facts about COVID-19 (2 items), symptoms (3 items), transmission (4 items), and treatment and prevention (5 items). Participants were given three options: Yes, Not sure, and No (1 = correct answer; 0 = wrong answer or not sure).

The Cronbach 's alpha value for the knowledge section was 0.637.

Section C assessed the respondents' attitude to COVID-19 using 8 questions, and each question used a 5 point Likert scale ranging from 1 = Strongly disagree to 5 = Strongly Agree. The total scores range from 0 to 40, with higher scores indicating a better attitude.

The Cronbach 's alpha value for the attitude section was 0.835.

Section D consists of 12 questions to evaluate respondents' preventive behavior. A 5 point Likert scale (1 = never; 2 = seldom; 3 = sometimes; 4 = often; 5 = always) was used to assess each item. All the scores were summed up and ranged from 12 to 60. A higher score indicated the participant practices better prevention behavior.

The Cronbach's alpha value for the preventive behavior section was 0.886.

Section E is the eHealth Literacy Scale (eHeaLS) (45) mainly to discern the participants' eHealth literacy level. This scale included 8 items with a 5-point Likert scale ranging from 1 = Strongly disagree to 5 = Strongly Agree. The total score ranged from 8 to 40 points (cut-off score ≥ 32). A higher score indicated a better literacy level.

The Cronbach's alpha value for the eHealth literacy section was 0.947.

### Statistical analysis

All analyses were carried out using IBM SPSS, version 21.0 (Chicago, IL, USA).

Frequencies and percentages are used to describe the distribution of categorical variables. Kolmogorov–Smirnov tests were used to verify that the data had a normal distribution. The Mann–Whitney *U* and Kruskal–Wallis tests were used to assess contributors to differences in knowledge, attitudes, and practices related to COVID-19. The correlations between variables were analyzed by Spearman's rank correlation test. Multinomial logistic regression was performed, to identify associated factors related to preventive behavior. A *P*-value < 0.05 was considered statistically significant.

## Results

### Sample characteristics

The sample characteristics of Anhui citizens and their eHealth literacy scores on COVID-19 were shown in Table 1. A total of 2,122 citizens (332 male and 1,790 female) in three regions of Anhui in China (155, 7.30% from the northern region, 306, 14.42% from the central region, and 1,661, 78.28% from the southern region) agreed to participate in the survey and complete the questionnaires assessing their knowledge, attitude, prevention behavior, and eHealth literacy regarding COVID-19 outbreaks. The response rate was 72.03%. The mean age of participants was 29.10 (SD 10.01) years, of which 26.20% were in aged 18–20 years old, 33.55% were aged 21–30 years old, 26.48% were aged 31–40 years old, and 10.23% were aged 41~50 years old. Of the sample, 65.22% were living in the city, 47.88% were single, 47.74% were married, and 4.38% were divorced or widowed. The education level of the sample comprised 3.49% middle school and below, 4.71% senior high school, 26.53% junior college, and 62.68% bachelor's. The majority (79.78%) of the participants were in good health; 64.75% were in a face-to-face working environment; 58.44% were afraid of infection with COVID-19; and 63.48% had family members who work in health care facilities. Every week, 33.27% of participants browsed the COVID-19 webpage <15 min, 34.21% for 15–30 min, and 19.75% for more than an hour.72.24% of participants reported having online COVID-19 lectures or training, 18.99% had injected the COVID-19 vaccine, 75.49% were at a high eHealth literacy level, and 5.28% had been exposed to suspected or confirmed COVID-19 patients (Table 1).

**Figure 1:**
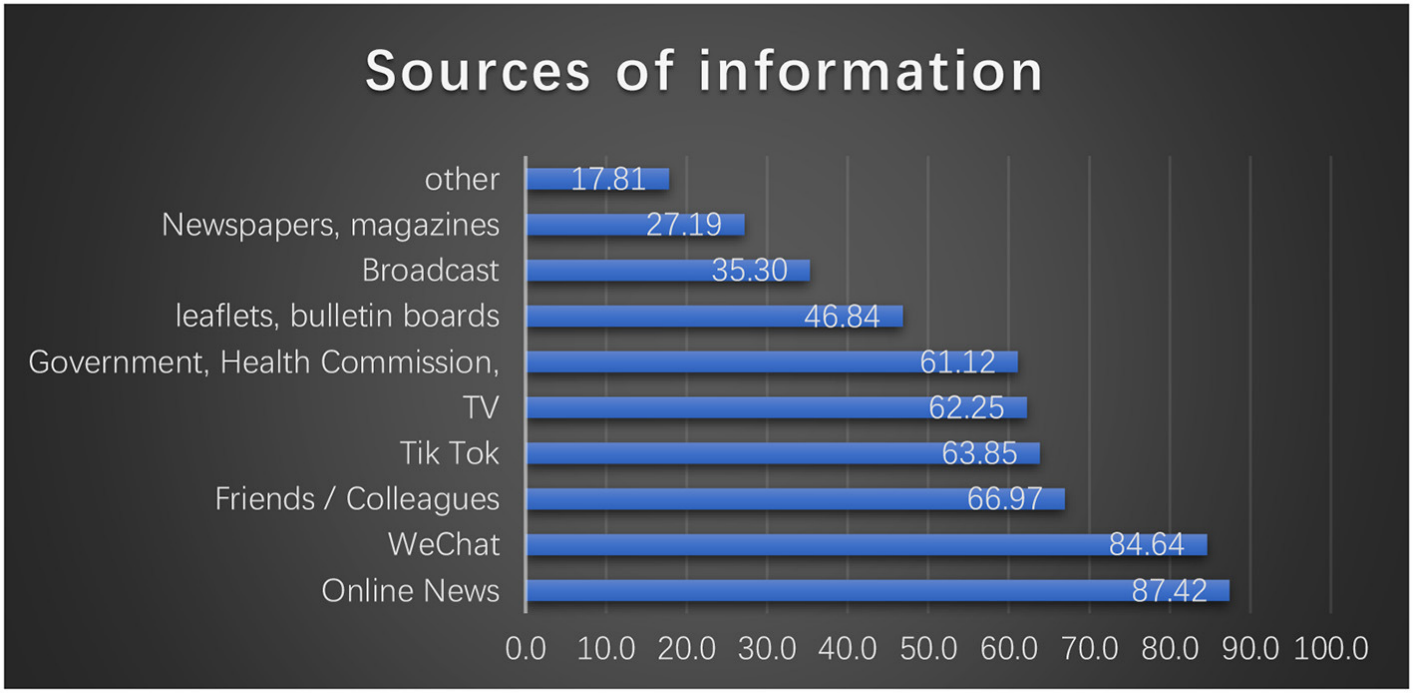
Source of COVID-19 information reported by citizens.

Anhui citizens are more likely to seek information about COVID-19 through online news, WeChat, friends or colleagues, TikTok, television, and information published by the government or health board (Figure 1). The mean (SD) scores of knowledge, preventive behavior, attitude, and eHealth literacy were 10.78 (1.89), 52.25 (8.27), 34.09 (4.24), and 34.23 (5.61), respectively.

### Knowledge of citizens about COVID-19

Most citizens were aware of the knowledge, including facts, symptoms, transmission, treatment, and prevention of COVID-19, as shown in Table 2. However, 35.06% of participants were not aware of the coronavirus that belongs to the β genus of coronaviruses. About 43.26% of participants held the misconception that people infected with COVID-19 were less likely to experience nasal congestion, runny nose, and sneezing with symptoms different from those of the common cold. Of the samples, 65.22% incorrectly believed that only people who are older or have underlying medical conditions will develop severe cases. 28.28% of citizens incorrectly responded that not all people are susceptible to COVID-19. Over 70% of citizens incorrectly believed that residents could wear general masks to prevent COVID-19 infection.

**Table 2:** Level of knowledge of COVID-19 (*N* = 2,122).

**Item**	**Correct answer**		**Incorrect answer**	
	***N***	**%**	***N***	**%**
**Fact**				
Q1. WHO announced on March 11, 2020 that the new coronary pneumonia (COVID-19) epidemic had been declared a global pandemic (pandemic) (T).	1,883	88.74	239	11.26
Q2. The new coronavirus belongs to the β genus of coronaviruses (T).	1,378	64.94	744	35.06
**Symptoms**				
Q3. The main clinical symptoms of COVID-19 are fever, fatigue, dry cough, shortness of breath, diarrhea, and myalgia (T).	1,994	93.97	128	6.03
Q4. Unlike with influenza, people infected with COVID-19 are less likely to have a stuffy nose, runny nose, and sneezing (T).	1,204	56.74	918	43.26
Q5. Only people who are older or with underlying health conditions will develop severe cases (F).	738	34.78	1,384	65.22
**Transmission**				
Q6. COVID-19 can be transmitted through human-to-human transmission (T).	2,089	98.44	33	1.56
Q7. Asymptomatic COVID-19-infected people are contagious (T).	1,956	92.18	166	7.82
Q8. COVID-19 is transmitted through air, aerosol, contact, foam and fecal pathways (T).	1,997	94.11	125	5.89
Q9. People are all susceptible to COVID-19 (T).	1,522	71.72	600	28.28
**Treatment and prevention**				
Q10. Vaccine available for COVID-19 (T).	1,918	90.39	204	9.61
Q11. Novel coronavirus pneumonia is a category B infectious disease and is managed as a category A infectious disease (T).	1,666	78.51	456	21.49
Q12. There is currently no effective treatment for COVID-19, but early symptomatic and supportive treatment can help most patients recover from infection (T).	1,934	91.14	188	8.86
Q13. People in contact with people infected with COVID-19 should be quarantined immediately for 14 days (T).	2,021	95.24	101	4.76
Q14. Residents can wear general masks to prevent COVID-19 infection (F).	569	26.81	1,553	73.19

The results of the knowledge score were significantly different in gender (Z_mwu_= −7.77, *P* < 0.001), living areas (Z_mwu_= −10.51, *P* < 0.001), family members who work in health care facilities (Z_mwu_= −10.89, *P* < 0.001), online COVID-19 lectures or training (Z_mwu_= −12.94, *P* < 0.001), vaccination with COVID-19 (Z_mwu_ = −8.75, *P* < 0.001), eHealth literacy (Z_mwu_ = −10.21, *P* < 0.001), working directly with people face-to-face (Z_mwu_ = −15.16, *P* < 0.001), exposure to suspected and confirmed COVID-19 patients (Z_mwu_ = −4.34, *P* < 0.001), age ($\chi_{\text{kw}}^{2}$ = 189.79, *P* < 0.001), education level ($\chi_{\text{kw}}^{2}$ = 50.11, *P* < 0.001), marital status ($\chi_{\text{kw}}^{2}$ = 123.61, *P* < 0.001), browsing the COVID-19 webpage time weekly ($\chi_{\text{kw}}^{2}$ = 53.40, *P* < 0.001), and region of Anhui ($\chi_{\text{kw}}^{2}$ = 101.33, *P* < 0.001) (Table 3).

**Table 3:** Level of knowledge, attitude, and prevention behavior according to demographic characteristics (*N* = 2,122).

**Variables**	**Characteristics**	***N***	**%**	**Knowledge**			**Practice**			**Attitude**			**eHealS**		
				**Mean rank**	**Z_mwu_**	***P***	**Mean rank**	**Z_mwu_**	***P***	**Mean rank**	**Z_mwu_**	***P***	**Mean rank**	**Z_mwu_**	***P***
Gender	Male	332	15.65	10657.51	−7.77	<0.001	8901.93	−24.71	<0.001	10890.30	−5.28	<0.001	10554.00	−8.70	<0.001
	Female	1,790	84.35	11572.61			11883.34			11531.41			11590.93		
Living areas	City	1,384	65.22	1160.76	−10.51	<0.001	1141.31	−8.31	<0.001	1116.23	−5.66	<0.001	1134.72	−7.70	<0.001
	Countryside	738	34.80	875.36			911.82			958.86			924.19		
Current health status	Bad	429	20.22	1034.00	−1.07	0.285	904.38	−6.01	<0.001	849.21	−8.07	<0.001	884.70	−6.84	<0.001
	Good	1,693	79.78	1068.47			1101.31			1115.29			1106.30		
Fear of infection with COVID-19	No	882	41.60	1071.11	−0.63	0.531	1105.24	−2.80	0.005^*^	1076.01	−0.92	0.355	1132.96	−4.63	<0.001
	Yes	1,240	58.40	1054.66			1030.39			1051.18			1010.67		
Have a family member working in a medical institution	No	775	36.52	875.70	−10.89	<0.001	876.88	−10.64	<0.001	963.34	−5.63	<0.001	932.10	−7.54	<0.001
	Yes	1,347	63.48	1168.40			1167.72			1117.98			1135.95		
Attend online COVID-19 lectures or training	No	589	27.76	791.39	−12.94	<0.001	774.56	−13.52	<0.001	934.29	−5.96	<0.001	830.87	−10.98	<0.001
	Yes	1,533	72.24	1165.28			1171.75			1110.38			1150.11		
Vaccination with COVID-19	No	1,719	81.01	1006.70	−8.75	<0.001	1021.98	−6.20	<0.001	1034.54	−4.21	<0.001	1025.38	−5.73	<0.001
	Yes	403	18.99	1295.24			1230.09			1176.49			1215.56		
eHealth literacy	Low	520	24.50	829.62	−10.21	<0.001	689.75	−16.10	<0.001	705.18	−15.34	<0.001	260.50	−35.06	<0.001
	High	1,602	75.50	1136.77			1182.17			1177.16			1321.50		
Face-to-face working environment	No	748	35.25	795.77	−15.16	<0.001	759.54	−16.93	<0.001	938.20	−6.87	<0.001	868.61	−10.93	<0.001
	Yes	1,374	64.75	1206.16			1225.88			1128.63			1166.51		
Exposure to suspected and confirmed COVID-19 patients	No	2,010	94.72	1048.26	−4.34	<0.001	1053.27	−2.65	0.008^*^	1057.50	−1.28	0.200	1051.18	−3.36	0.001^**^
	Yes	112	5.28	1299.09			1209.14			1133.36			1246.69		
Age	18~20	556	26.20	796.32	189.78	<0.001	852.55	101.03	<0.001	959.82	30.99	<0.001	919.45	75.58	<0.001
	21~30	712	33.55	1071.38			1107.34			1054.53			1090.36		
	31~40	562	26.48	1280.91			1200.52			1160.23			1207.18		
	41~50	217	10.23	1105.67			1101.61			1094.66			1024.16		
	≥50	75	3.53	1161.63			1017.57			1045.70			856.99		
Education level	Middle school and below	74	3.49	658.38	50.11	<0.001	816.50	70.29	<0.001	1068.05	14.06	<0.001	695.27	39.67	<0.001
	Senior high school	100	4.71	852.60			792.35			976.81			885.85		
	Junior college	563	26.53	1094.49			1201.60			1135.61			1074.70		
	Bachelor	1,330	62.68	1083.52			1045.30			1041.35			1086.24		
	Master and above	55	2.59	1113.60			838.22			935.45			1140.19		
Marital status	Single	1,016	47.88	915.53	123.61	<0.001	958.69	59.12	<0.001	995.76	23.50	<0.001	983.50	37.02	<0.001
	Married	1,013	47.74	1209.60			1165.59			1126.97			1144.13		
	Divorced/Others	93	4.38	1043.03			1050.94			1066.65			1013.60		
Browse the COVID-19 webpage time weekly	<15 min	706	33.27	940.70	45.53	<0.001	845.06	159.14	<0.001	927.30	73.67	<0.001	895.42	107.08	<0.001
	15–30 min	726	34.21	1105.10			1120.34			1069.59			1083.84		
	30–60 min	271	12.77	1112.47			1111.78			1105.97			1114.74		
	>60 min	419	19.75	1156.52			1291.72			1244.85			1268.20		
Region of Anhui	Northern	155	7.30	804.37	101.33	<0.001	821.57	84.05	<0.001	987.12	7.33	0.026^*^	888.87	38.66	<0.001
	Central	306	14.42	819.46			838.20			996.89			918.22		
	Southern	1,661	78.28	1130.08			1125.03			1080.34			1104.00		

** *P* < 0.01.

* *P* < 0.05.

### The attitude of citizens toward COVID-19

Nearly 80–90% of the participants agreed that injecting the COVID-19 vaccines, staying at home during the Spring Festival, and not returning to their hometown without necessity, strengthening self-protection, and health education would drive down transmission. Only 52.16% of the population agreed that the targets for terminating the coronavirus pandemic would be achieved this year. Active cooperation with the government's epidemic prevention and control work has been supported by almost all citizens (96.52%), although the epidemic prevention and control measures would affect their work and daily life. The majority of participants had an acceptable attitude (94.07%) that SARS-CoV-2 nucleic acid testing should be performed for those who had visited a high-risk county (district) in COVID-19 within 14 days (Table 4).

**Table 4:** Attitude toward COVID-19 (*N* = 2,122).

**Items**	**Mean**	**SD**	**Strongly** **disagree**	**Partly** **disagree**	**Neutral**	**partly** **Agree**	**Strongly** **agree**
			***N*** **(%)**	***N*** **(%)**	***N*** **(%)**	***N*** **(%)**	***N*** **(%)**
A1. COVID-19 is more dangerous than SARS and H7N9.	4.11	0.93	23 (1.08)	116 (5.47)	327 (15.41)	786 (37.04)	870 (41.00)
A2. I believe that the COVID-19 epidemic can be terminated this year.	3.69	0.94	25 (1.18)	107 (5.04)	883 (41.61)	583 (27.47)	524 (24.69)
A3. The vaccine plays an important role in controlling epidemics.	4.22	0.72	3 (0.14)	15 (0.71)	299 (14.09)	992 (46.75)	813 (38.31)
A4. Staying at home during the Spring Festival and not returning home without necessity will help control COVID-19.	4.43	0.71	17 (0.80)	27 (1.27)	89 (4.19)	890 (41.94)	1,099 (51.79)
A5. Increased self-protection can protect against COVID-19 infection.	4.18	0.84	13 (0.51)	84 (3.96)	250 (11.78)	926 (43.64)	849 (40.01)
A6. Health education plays an important role in the prevention and control of COVID-19.	4.46	0.61	7 (0.33)	10 (0.47)	61 (2.87)	969 (45.66)	1,075 (50.66)
A7. I will actively cooperate with the epidemic prevention and control work, although the government's epidemic prevention and control measures will affect my work and daily life.	4.52	0.61	7 (0.33)	11 (0.52)	56 (2.64)	850 (40.06)	1,198 (56.46)
A8. A nucleic acid test should be performed for people with a 14-day history of travel in a county (district) at moderate to high risk.	4.47	0.76	24 (1.13)	48 (2.26)	54 (2.54)	784 (36.95)	1,212 (57.12)

There was a significant difference in attitude based on gender (Z_mwu_= −5.28, *P* < 0.001), living areas (Z_mwu_ = −5.66, *P* < 0.001), health status (Z_mwu_ = 8.07, *P* < 0.001), family members who work in health care facilities (Z_mwu_ = −5.63, *P* < 0.001), online COVID-19 lectures or training (Z_mwu_ = −5.96, *P* < 0.001), vaccination with COVID-19 (Z_mwu_ = −4.21, *P* < 0.001), eHealth literacy (Z_mwu_ = −15.34, *P* < 0.001), working directly with people face-to-face (Z_mwu_ = −6.87, *P* < 0.001), age ($\chi_{\text{kw}}^{2}$ = 30.99, *P* < 0.001), education level ($\chi_{\text{kw}}^{2}$ = 14.06, *P* < 0.001), marital status ($\chi_{\text{kw}}^{2}$ = 23.50, *P* < 0.001), browsing the COVID-19 webpage time weekly ($\chi_{\text{kw}}^{2}$ = 73.67, *P* < 0.001), and region of Anhui ($\chi_{\text{kw}}^{2}$ = 7.33, *P* < 0.05) (Table 3).

### eHealth literacy of Anhui citizens

Approximately 75.50 % of participants had a high level of eHealth literacy. There was a significant difference in eHealth literacy according to gender (Z_mwu_ = −8.70, *P* < 0.001), living areas (Z_mwu_ = −7.70, *P* < 0.001), health status (Z_mwu_ = −6.84, *P* < 0.001), fear of infection with COVID-19 (Z_mwu_ = −4.63, *P* < 0.001), family members who work in health care facilities (Z_mwu_ = −7.54, *P* < 0.001), online COVID-19 lectures or training (Z_mwu_ = −10.98, *P* < 0.001), vaccination with COVID-19 (Z_mwu_ = −5.73, *P* < 0.001), eHealth literacy (Z_mwu_ = −35.06, *P* < 0.001), work with people face-to-face directly (Z_mwu_ = −10.93, *P* < 0.001), exposure to suspected and confirmed COVID-19 patients (Z_mwu_ = −3.36, *P* < 0.01), age ($\chi_{\text{kw}}^{2}$ = 75.58, *P* < 0.001), education level ($\chi_{\text{kw}}^{2}$ = 39.67, *P* < 0.001), marital status ($\chi_{\text{kw}}^{2}$ = 37.02, *P* < 0.001), browse the COVID-19 webpage time weekly ($\chi_{\text{kw}}^{2}$ = 107.08, *P* < 0.001), and region of Anhui ($\chi_{\text{kw}}^{2}$ = 38.66, *P* < 0.001) (Table 3).

### Prevention behavior of citizens about COVID-19

The table shows that the majority of citizens were able to implement preventive behaviors. The responses to the preventive behavior questions reported that 14.56, 13.86, 13.43, 12.54, 11.97, and 11.97% of the citizens had never or rarely performed the items 1, 3, 4, 6, 11, and 12, respectively (Table 5).

**Table 5:** Level of preventive behavior for COVID-19 (*N* = 2,122).

**Item (in the past week)**	**Never**	**Seldom**	**Sometimes**	**Often**	**Always**
	***N*** **(%)**	***N*** **(%)**	***N*** **(%)**	***N*** **(%)**	***N*** **(%)**
B1. Reduce outings, social activities and avoid congested areas	126 (5.94)	183 (8.62)	145 (6.83)	365 (17.20)	1,303 (61.40)
B2. Wear a mask when going out	11 (0.52)	70 (3.30)	60 (2.83)	272 (12.82)	1,709 (80.54)
B3. Temperature monitoring	35 (1.65)	259 (12.21)	170 (8.01)	336 (15.83)	1,322 (62.30)
B4. Avoid direct contact with potentially infected public facilities, such as elevator buttons and stair railings:	53 (2.50)	232 (10.93)	227 (10.70)	461 (21.72)	1,149 (54.15)
B5. Cover your mouth with a paper towel or other tissue when coughing or sneezing.	24 (1.13)	133 (6.27)	144 (6.79)	370 (17.44)	1,451 (68.38)
B6. Maintain a social distance of more than one meter	18 (0.85)	248 (11.69)	306 (14.42)	506 (23.85)	1,044 (49.20)
B7. Use soap to wash your hands frequently.	19 (0.90)	127 (5.98)	169 (7.96)	386 (18.19)	1,421 (66.97)
B8. Homes are often disinfected and ventilated,	7 (0.33)	94 (4.43)	142 (6.69)	453 (21.35)	1,426 (67.20)
B9. Proactive presentation of real-time health codes is not forged by cell phone screenshots	46 (2.17)	40 (1.89)	54 (2.54)	194 (9.14)	1,788 (84.26)
B10. Do not consume imported cold chain foods and other fruits found positive for nucleic acid (e.g., Cherries, etc.).	91 (4.29)	107 (5.04)	153 (7.21)	309 (14.56)	1,462 (68.90)
B11. Do not touch your eyes, nose, or mouth with unwashed hands (even if you are wearing a mask).	62 (2.92)	192 (9.05)	253 (11.92)	411 (19.37)	1,204 (56.74)
B12. When dining out, insist on “one dish with one common chopstick and one soup with one common spoon.”	44 (2.07)	210 (9.90)	225 (10.60)	466 (21.96)	1,177 (55.47)

The results of the prevention behavior score differed significantly by gender (Z_mwu_ = −24.71, *P* < 0.001), living areas (Z_mwu_ = −8.31, *P* < 0.001), health status (Z_mwu_ = −6.01, *P* < 0.001), fear of infection with COVID-19 (Z_mwu_ = −2.80, *P* < 0.001), family members who work in health care facilities (Z_mwu_ = −10.64, *P* < 0.001), attending online COVID-19 lectures or training (Z_mwu_ = −13.52, *P* < 0.001), vaccination with COVID-19 (Z_mwu_ = −6.20, *P* < 0.001), eHealth literacy (Z_mwu_ = −16.10, *P* < 0.001), working with people face-to-face directly (Z_mwu_ = −16.93, *P* < 0.001), exposure to suspected and confirmed COVID-19 patients (Z_mwu_ = −2.65, *P* < 0.01), age ($\chi_{\text{kw}}^{2}$ = 101.03, *P* < 0.001), education level ($\chi_{\text{kw}}^{2}$ = 70.29, *P* < 0.001), marital status ($\chi_{\text{kw}}^{2}$ = 59.12, *P* < 0.001), browsing the COVID-19 webpage time weekly ($\chi_{\text{kw}}^{2}$ = 159.14, *P* < 0.001), and region of Anhui ($\chi_{\text{kw}}^{2}$ = 84.05, *P* < 0.001) (Table 3).

### Influencing factors on preventive behavior for COVID-19

The category classification of preventive behaviors was used as the dependent variable, and a multivariate logistic regression analysis was conducted with the significant factors in the univariate analysis as independent variables.

In the multiple logistic regression analysis, the scores of preventive behaviors were divided into 3 categories, namely rarely group (score < 60), sometimes group (60 ≤ score < 80), and always group (score ≥ 80). The third group (always) was used as the reference group.

Compared with the group of citizens that always exhibit preventive behaviors, citizens who had a poor attitude (OR = 0.782, *P* < 0.001), had low eHealth literacy (OR = 0.875, *P* < 0.001), male (OR = 3.170, *P* < 0.001), have a worse health status (OR = 2.417, *P* < 0.05), do not have family members working in health care facilities (OR = 1.882, *P* < 0.05), and do not work in a face-to-face environment (OR = 2.598, *P* < 0.05) rarely exhibit preventive behaviors. Meanwhile, those who sometimes took preventive behaviors were more likely to be lacking in knowledge on COVID-19 (OR = 0.892, *P* < 0.05), to have a negative attitude toward COVID-19 (OR = 0.872, *P* < 0.001), to have a low eHealth literacy (OR = 0.916, *P* < 0.001), to be male (OR = 1.490, *P* < 0.05), to have a poor health status (OR = 1.393, *P* < 0.05), to have not participated in COVID-19 lectures or training (OR = 0.710, *P* < 0.05), to not work in a face-to-face environment (OR = 2.598, *P* < 0.001), to have a junior high school education level or less (OR = 0.382, *P* < 0.05), and to browse the COVID-19 webpage time < 15 min weekly (OR = 1.511, *P* < 0.05), than those were in the always group (Table 6).

**Table 6:** Multinomial logistic regression analysis of the factors affecting preventive behavior level toward COVID-19 among the nursing students (*N* = 2,122).

**Item**		**Seldom**			**Sometime**		
		**OR**	**95%CI**	***P***	**OR**	**95%CI**	***P***
Knowledge	Knowledge	0.916	0.818, 1.026	0.130	0.892	0.834, 0955	0.001^**^
Attitude	Attitude	0.782	0.732, 0.835	<0.001	0.872	0.841, 0.904	<0.001
eHealth literacy	eHealth literacy	0.875	0.836, 0.917	<0.001	0.916	0.894, 0940	<0.001
Age	Age	0.977	0.938, 1.017	0.252	0.997	0.978, 1.016	0.758
Gender	Male	3.170	1.832, 5.487	<0.001	1.490	1.077, 2.062	0.016^*^
	Female	Ref			Ref		
Living areas	City	0.784	0.451, 1.365	0.390	0.941	0.703, 1.259	0.680
	Rural	Ref			Ref		
Health status	Bad	2.417	1.416, 4.127	<0.001	1.393	1.037, 1.872	0.028^*^
	Good	Ref			Ref		
Fear of infection	No	0.973	0.588, 1.608	0.914	0.832	0.646, 1.071	0.153
	Yes	Ref			Ref		
Have a family member working in a medical institution	No	1.882	1.004, 3.528	0.048^*^	1.120	0.834, 1.504	0.452
	Yes	Ref			Ref		
Attend online COVID-19 lectures or training	No	0.740	0.417, 1.312	0.303	0.710	0.528, 0.953	0.023^*^
	Yes	Ref			Ref		
Vaccination	No	0.509	0.209, 1.236	0.136	0.764	0.528, 1.105	0.153
	Yes	Ref			Ref		
Face-to-face working environment	No	2.598	1.175, 5.744	0.018^*^	2.279	1.589, 3.269	<0.001
	Yes	Ref			Ref		
Exposure to suspected and confirmed COVID-19 patients	No	0.195	0.021, 1.811	0.151	1.532	0.876, 2.679	0.135
	Yes	Ref			Ref		
Education level	Middle school and below	1.031	0.170, 6.252	0.973	0.382	0.152, 0.960	0.041^*^
	Senior high school	2.666	0.481, 14.769	0.262	0.607	0.263, 1.401	0.242
	Junior college	0.852	0.157, 4.609	0.852	0.422	0.206, 0.865	0.019
	Bachelor	0.882	0.179, 4.340	0.878	0.550	0.281, 1.075	0.080
	Master and above	Ref			Ref		
Marital status	Single	1.294	0.418,4.008	0.655	1.119	0.616, 2.035	0.712
	Married	2.690	0.665, 10.872	0.165	1.042	0.540, 2.009	0.903
	Divorced/others	Ref			Ref		
Browse the COVID-19 webpage time weekly	<15 min	1.770	0.755, 4.153	0.189	1.511	1.037, 2.201	0.031^*^
	15–30 min	0.909	0.364, 2.267	0.837	1.090	0.745, 1.596	0.657
	30–60 min	0.986	0.332, 2.927	0.980	0.960	0.594, 1.550	0.866
	>60 min	Ref			Ref		
Region of Anhui	Northern	1.618	0.725, 3.613	0.240	0.639	0.391, 1.045	0.074
	Central	1.607	0.850,3.037	0.144	0.936	0.655, 1.337	0.717
	Southern	Ref			Ref		

** *P* < 0.01.

* *P* < 0.05.

## Discussion

Although COVID-19 has shifted from a pandemic response model to coexistence with the virus, ongoing public health measures are essential. This is because some countries and regions still face localized spikes or localized recurrences of large numbers of confirmed and fatal cases. Moreover, in the second year of a COVID-19 pandemic, following prevention guidelines and directions may be difficult due to pandemic fatigue. Therefore, it remains important to understand citizens' preventive behaviors for COVID-19.

In China, online news (87.4%) and WeChat (84.6%) are today's important health information resources. In North-Central Nigeria, the internet, social media (55.7%), and television (27.5%) were the main information resources. According to the Office for National Statistics, in the UK, 59% of females and 50% of males have accessed health-related information online within 3 months (46). The Internet and social media are broadly recognized as a health communication and education instrument for transforming medical care and public health in Italy (47–49). Internet-based health information and knowledge may be a valuable resource for health-behavior interventions and programs (50, 51). Following such research, the means of information dissemination should be utilized to improve COVID-19 epidemic stewardship. A wide range of interventions could be designed to help individuals access COVID-19 information by developing public eHealth literacy, training health information professionals to provide sustained conversation services online, and disseminating quality health information to the general population (48, 52). The government and health agencies should make an effort to inform citizens about which information channels or websites to use and to provide them with specific assistance (53). This will prevent citizens from being misled by misinformation. Additionally, another study conducted in Ethiopia showed that public information is primarily obtained through television and radio (72.6%) (54). During such tough times, it is still necessary to assist the underprivileged population who lack access to information on official online platforms due to the lack of basic infrastructure.

In this study, participants demonstrated a high level of knowledge about SARS-CoV-2 infection, positive attitudes, acceptable practices, and a good level of eHealth literacy.

Through the combined efforts of government authorities and healthcare workers, 1 year since the COVID-19 pandemic, the knowledge and awareness of controlling the spread of infection has improved. Our results show that the participants had sufficient knowledge regarding symptoms, transmission, and treatment of COVID-19. However, lack of knowledge about virus types (35.06%), virus management classifications (21.49%), susceptible populations (28.28%), populations developing severe cases (65.22%), similarities and differences between influenza and COVID-19 (43.26%), and the choice of type of mask to wear (73.19%), may still be widespread.

Concerning attitudes, the results of the study showed that most of the participants had positive attitudes and were actively cooperating with the government in COVID-19 prevention. However, nearly half of the participants lack confidence in epidemic control and termination. This finding was contrary to the study conducted among the community health workers in Nepal (55). A total of ~85.06% of respondents stated that COVID-19 vaccines can protect recipients from infection by building up immunity. Thus, perceived COVID-19 severity and vaccine benefits help the government to deal with vaccine hesitancy and achieve high vaccination coverage rates, which was supported by a study in Hong Kong (56).

Factors influencing the residents' practice of COVID-19 were assessed. Adequate knowledge and positive attitudes are the driving forces for more feasible and effective behavior, which are supported by the principle of the KAP model (57–59). The eHealth literacy score showed a positive independent association with adherence to protective behaviors. Similar findings have been demonstrated in previous studies that participants with lower eHealth literacy scores were less likely to seek health information (60, 61). Some studies have reported the relationship between eHealth literacy scores and health outcomes. Minh H. Nguyen et al. noted that the fear of COVID-19 can be mitigated and health-related quality of life can be improved as a result of eHealth literacy (34). The Australian survey showed that respondents who received higher eHealth literacy scores would perform “critically appraisal” information on the Internet (62).

A study by Lawrence An, USA, found that people with higher CoV-eHealth literacy scores had a greater rejection of conspiracy theories (37). Strengthening eHealth literacy is seen as a way to curb the spread of the pandemic and improve the behavior of the general population.

It was observed that male respondents adopted fewer safety practices than females. The studies from Iran, and Saudi Arabia also exhibit a similar result (34, 63). Much of the research showed that females had a higher rate of compliance with preventive behaviors (37, 62). Also, published literatures show that women are more prone to having varied fears related to COVID-19, such as health, economic, and political crises caused by pandemics, and have higher COVID-19 stress exposure (64–69). Individuals who perceived the severity of COVID-19 were more likely to comply with COVID-19 prevention measures (53). Moreover, previous studies found that men are more likely to engage in risk-taking behavior (59, 70). Therefore, targeting men, implementing public awareness campaigns, community engagement strategies, and health education programs are recommended (59, 71).

The study found that healthier residents were more likely to take preventive precautions than those in poorer health. Evidence to support the conclusion has been drawn from research on health belief models (HBM) and preventive health behavior (72, 73). HBM is a theoretical model used to guide health promotion, explain and predict health behaviors, and disease prevention. Based on the Health Belief Model (HBM), in order to prevent infection with COVID-19, the intention to carry out preventive activities will increase by the healthier person. This could imply that increased awareness of COVID-19 information may predispose people to take effective precautionary measures.

As for family members of health care workers (HCWs), the practice score of the participants was reported to be higher. This was supported by previous studies that showed good infection control practices were observed among HCWs in relation to COVID-19 (74). HCWs may have a direct effect on the practices of family members, and, consequently, the citizens adopt more proactive preventive measures.

The current survey found that despite not having been trained or lectured on COVID-19, participants had a higher level of practice than expected. The study also showed that citizens with a middle school education or less took more protective measures than those with a master's degree or higher. The main reason for this was that these adults with lower secondary education and no training or lectures might maintain social lives through manual labor. Also, the prevalence of COVID-19 infections in China and worldwide has been reported daily in the media, making people feel more threatened about being infected. Under these circumstances, they may fear being quarantined and isolated due to infection, which could result in a loss of income and productivity. In addition, as the government encourages companies and social organizations to undertake health management, the growing health concern has created a more favorable environment for conducting preventive behavior. Individuals with a green health code are free in public spaces, whereas those with a yellow or red code are barred from public places, public transportation stations, and residential areas by security guards and gatekeepers (43, 44). The implementation of the Health Code has created an efficient way to respond to the COVID-19 pandemic (75).

This study's finding showed that participants in face-to-face work environments exhibit better practice. According to previous research, people who work face-to-face experience more financial threats, especially those with caregiving responsibilities (76). And for social-health personnel (such as doctors and nurses), professional face-to-face activity had to be continued, because it was deemed essential. For occupational reasons, employees who work in a face-to-face modality may be potentially exposed to COVID-19. Despite the high risks faced by individuals, they may place great emphasis on transmission risk mitigation, so the level of adherence to COVID-19 effective preventive measures was high. Another study showed that Latino and black frontline workers in high-risk occupations were less likely to take adequate COVID-19 protection (77). These differences may be related to sociodemographic conditions, countries' response patterns, and cultural differences.

Based on the findings of this study, a lower practice score was observed in people who spent time on the COVID-19-related media for <15 min, compared with people who consumed more than 1 h. It may be because the fact that COVID-19 information may cause intense concern about infection, which would increase protective behavior (78). However, another study (79) in the US noted that increased exposure to social media to learn about COVID-19 increased mental distress, and reduced the likelihood of compliance with health guidance measures as a result. This discrepancy might be due to higher information overload in Western populations (80). Multiple studies have shown that misinformation and conspiracy theories could reduce health behaviors. Therefore, evidence-based information should be disseminated by the public health authorities, such as the governments, the Centers for Disease Control, and the World Health Organization. It is also important to increase individuals' eHealth literacy, which helps them to examine information credibility.

### Strengthen and limitations of the study

To our knowledge, this was one of the first studies on empirical evidence on the COVID-19 related knowledge, attitudes, and practices (KAP) and eHealth literacy in the second year of the pandemic of residents in China.

This study had some limitations. First, the online study was self-reported, which could exist the recall bias and social desirability bias. Thus, face-to-face interviews among vulnerable populations (e.g., older adults) who lack basic infrastructure also deserve special study. Second, due to the cross-sectional survey conducted in one province in China, the generalizability might be compromised, and causal relationships cannot be established. Third, the participants were overwhelmingly composed of females. Therefore, this result may be limited to a lack of sufficient male cases.

## Conclusions

In this study, the residents in China acquired good knowledge, formed positive attitudes, performed the appropriate preventive behavior toward COVID-19 and had basic eHealth literacy. It is vital for the government and health agencies to inform citizens concerning which information channels or websites to use and assist the underprivileged population who lack basic infrastructure. In particular, the information on the type of mask to wear and populations developing severe cases still needs to be reinforced by the Chinese National Health Council and the governments.

Our findings suggest that good knowledge, positive attitudes, and enhanced eHealth literacy were seen as a way to improve the behavior of the population. Implementing public awareness campaigns, community engagement strategies, and health education programs that target the male gender may improve men's preventive behavior. Based on HBM, people in good health, family members with HCW, being in a face-to-face work environment, and viewing COVID-19-related information for a long time may predispose people to take effective precautionary measures. The implementation of the Health Code may facilitate more protective measures for those with a middle school education or less, and those who have not received COVID-19 training or lectures.

## Data availability statement

The original contributions presented in the study are included in the article/supplementary material, further inquiries can be directed to the corresponding author/s.

## Ethics statement

Written informed consent was obtained from the individual(s) for the publication of any potentially identifiable images or data included in this article.

## Author contributions

TY, HL, and XDL conceived the study and performed the statistical analysis. MZ, XBT, and SJX carried out the literature searches. XBT, SJX, and HL distributed the online questionnaire and extracted the data. XBT assessed the study quality. TY and XDL wrote the manuscript. TY, HL, MZ, XDL, XBT, and SJX revised the manuscript. All authors contributed to the concept of this study. All authors read the published version of the manuscript and gave their consent.

## Funding

This work was supported by Anhui Provincial Department of Education College Outstanding Talent Cultivation Funding Project, No. gxgwfx2019032; the Teaching Quality and Teaching Reform Project of Anhui Provincial Department of Education, No. 2020jyxm2090; and Humanities and Social Sciences Research Project of Colleges and Universities in Anhui Province (SK2018A0199).

## Conflict of interest

The authors declare that the research was conducted in the absence of any commercial or financial relationships that could be construed as a potential conflict of interest.

## Publisher's note

All claims expressed in this article are solely those of the authors and do not necessarily represent those of their affiliated organizations, or those of the publisher, the editors and the reviewers. Any product that may be evaluated in this article, or claim that may be made by its manufacturer, is not guaranteed or endorsed by the publisher.

## Abbreviations

PHEIC: public health emergency
HCWs: health care workers
eHeals: the eHealth Literacy Scale
KAP, knowledge: attitudes and practices.
